# Identification and Validation of Chromobox Family Members as Potential Prognostic Biomarkers and Therapeutic Targets for Human Esophageal Cancer

**DOI:** 10.3389/fgene.2022.851390

**Published:** 2022-04-06

**Authors:** Xuefen Fang, Junjun Wang, Jiabing Chen, Mingkai Zhuang, Tingxuan Huang, Zhixin Chen, Yuehong Huang, Biyun Zheng, Xiaozhong Wang

**Affiliations:** ^1^ Department of Gastroenterology and Fujian Institute of Digestive Disease, Fujian Medical University Union Hospital, Fuzhou, China; ^2^ Fujian Medical University Cancer Center, Fujian Medical University, Fuzhou, China; ^3^ Department of Clinical Laboratory, Fujian Provincial Hospital Southern Branch, Fuzhou, China; ^4^ Department of Endoscopy Center, Fudan University Shanghai Cancer Center, Shanghai, China

**Keywords:** chromobox (CBX) protein, esophageal cancer, bioinformatics analysis, prognosis, biomarker, immunofluorescence

## Abstract

**Background:** Chromobox family proteins (CBXs) are vital components of epigenetic regulation complexes and transcriptionally inhibit target genes by modifying the chromatin. Accumulating evidence indicates that CBXs are involved in the initiation and progression of multiple malignancies. However, the expression, function, and clinical relevance such as the prognostic and diagnostic values of different CBXs in esophageal carcinoma (ESCA) are still unclear.

**Methods:** We applied Oncomine, TCGA, GEO, GEPIA, UALCAN, Kaplan–Meier plotter, cBioPortal, Metascape, and TIMER to investigate the roles of CBX family members in ESCA. Additionally, quantitative real-time PCR (RT-PCR), western blot, and immunofluorescence were used to verify the expression of CBX family members in ESCA clinical samples.

**Results:** Compared with normal tissues, the mRNA expression levels of CBX1/3/8 were significantly increased in ESCA, whereas CBX7 mRNA expression was reduced in both the TCGA cohort and GEO cohort. In the TCGA cohort, ROC curves suggested that CBX1/2/3/4/8 had great diagnostic value in ESCA, and the AUCs were above 0.9. Furthermore, upregulation of CBX1/3/8 and downregulation of CBX7 were closely related to the clinicopathological parameters in ESCA patients, such as tumor grades, tumor nodal metastasis status, and TP53 mutation status. The survival analysis indicated that higher CBX1/3/8 mRNA expressions and lower CBX7 expression suggested an unfavorable prognosis in ESCA. High genetic change rate (52%) of CBXs was found in ESCA patients. Functions and pathways of mutations in CBXs and their 50 frequently altered neighbor genes in ESCA patients were investigated; the results showed that DNA repair and DNA replication were correlated to CBX alterations. Moreover, we found a significant correlation between the expression level of CBX family members and the infiltration of immune cells in ESCA. Finally, we verified the expression of CBX family members in clinical samples and found the results were consistent with the databases.

**Conclusion:** Our study implied that CBX1/3/7/8 are potential targets of precision therapy for ESCA patients and new biomarkers for the prognosis.

## Introduction

ESCA is the seventh most frequent cancer and the sixth leading cause of cancer death, which accounts for almost 572,000 new cases and more than 509,000 deaths annually (Bray et al., 2018). Although prominent progress in the diagnosis and treatment of ESCA has been achieved, clinical outcomes for ESCA patients remain dismal, with 15–25% five-year overall survival rate worldwide (Pennathur et al., 2013). Therefore, the management of ESCA patients remains a considerable therapeutic challenge. It is extremely urgent to investigate the underlying mechanism of the carcinogenesis of ESCA, which will provide novel insights into the discovery of innovative therapeutic targets and diagnostic biomarkers.

Increasing evidence has suggested that aberrant epigenetic regulation influences the pathogenesis and progression of ESCA (Cao et al., 2020). The Polycomb group (PcG) complex, involved in the important epigenetic mechanism that regulates gene expression through chromatin mollification, plays principal roles in maintaining stem cell pluripotency and senescence and is implicated in cancer and other diseases (Chan and Morey, 2019). PcG complexes can be assembled into two distinct multi-protein complexes known as Polycomb Repressive Complex 1 and 2 (PRC1 and PRC2), which are associated with gene silencing via histone-modifying activities (Morey and Helin, 2010; Chan and Morey, 2019). As canonical components of PRC1, CBX family members have been shown to control the tumorigenesis and progression of several human malignancies by increasing the tumor stem cells’ self-renewal (Klauke et al., 2013; Gil and O'Loghlen, 2014).

Until now, a total of eight members of the CBX family (CBX1/2/3/4/5/6/7/8) have been identified in the human genome, with links mainly to heterochromatin, gene expression, apoptosis, and developmental program (Kaustov et al., 2011; Ma et al., 2014). CBXs are categorized into two types based on their molecular structure: 1) heterochromatin protein 1 (HP1) includes CBX1, CBX3, and CBX5 that share an N-terminal chromosome and a C-terminal chromophore domain; 2) polycomb (Pc) includes CBX2, CBX4, CBX6, CBX7, and CBX8 with a conserved N-terminal chromodomain and a C-terminal polycomb repressor box (Wotton and Merrill, 2007).

Deregulation of CBXs is associated with tumorigenesis of various cancer types and has significant prognostic value. Multi-omics integrative analysis revealed the antagonistic roles of CBX2 and CBX7 in metabolic reprogramming of breast cancer, which could predict patients’ outcomes and sensitivity to FDA-approved/investigational drugs (Iqbal et al., 2021). In hepatocellular carcinoma (HCC), aberrant expressions of eight CBXs members were significantly associated with clinical cancer stages and pathological tumor grades (Ning et al., 2018). Higher mRNA expressions of CBX1/2/3/6/8 were related to shorter overall survival (OS) in HCC patients (Ning et al., 2018). Recent reports have indicated that higher mRNA expression of CBX3-6 and lower mRNA expression of CBX7 were significantly associated with poor prognosis and survival rate of gastric cancer patients (Ma et al., 2020). Upregulation of CBX2 in patients with ESCA was intensely related to poor disease-specific survival and recurrence rate (Ueda et al., 2020). Overexpression of CBX8 in ESCA was correlated with cell proliferation and predicted poor prognosis (Zhang et al., 2019). Conversely, it was reported that CBX8 could directly suppress the Snail promoter activity, contributing to inhibiting ESCA metastasis (Wang et al., 2017). CBXs may play both anti-tumor and pro-tumor roles depending on tumor types and cellular context. Therefore, it is necessary to clarify profoundly the distinct functions and prognostic value of CBX family members in ESCA.

To the best of our knowledge, this was the first study conducted to explore the potential oncogene values of CBX family members in ESCA using integration bioinformatics analysis. Due to the rapid development of microarray technology and RNA-sequencing technology in the last decade, RNA and DNA research has taken a great revolution and become an essential component of biomedical research (Sealfon and Chu, 2011). In this regard, we analyzed the expression, clinical parameters, and genetic alterations of different CBX proteins in ESCA patients and predicted their prognostic values, utilizing thousands of gene expression or copy number variations published online. Overall, our results indicated that CBXs serve as effective prognostic biomarkers and potential targets for the research on the clinical intervention of ESCA.

## Materials and Methods

### ONCOMINE

ONCOMINE (www.oncomine.org) is an accessible online cancer microarray database providing powerful, genome-wide expression analysis (Rhodes et al., 2004). In our study, data were obtained to assess the transcriptional expression of CBX proteins between different carcinomas and adjacent normal control tissues ESCA. The difference in transcriptional expression of CBXs in ESCA was determined by Student’s t-test. Sufficient fold changes ≥1.5, significant *p* value < 0.05, and gene rank ≥ the top 10% were set as the threshold.

### The Cancer Genome Atlas (TCGA) Database

We obtained the raw counts of RNA-sequencing data and corresponding clinical information of CBX family members of TCGA ESCA tissue data from the TCGA dataset (http://tcga-data.nic.nih.gov/). RNAseq data in FPKM (Fragments Per kilobase per Million) format and log2 conversion for expression comparison between samples (Vivian et al., 2017). We draw receiver-operating characteristic (ROC) curves by using the “pROC” package. For Kaplan–Meier curves, *p* values and the hazard ratio (HR) with 95% confidence interval (CI) were generated by log-rank tests and unvaried Cox proportional hazards regression. CBX family member expressions and their correlation with the infiltration abundance of immune cells such as ADC [activated DC], B cells, CD8 T cells, Cytotoxic cells, DC, Eosinophils, and iDC [immature DC] in ESCA were evaluated using Spearman’s correlation with TCGA ESCA in the project level 3 HTSeq-RNAseq FPKM format data and clinical data. All analytical methods previously mentioned and R packages were performed using R software version v3.3.6 (Liu et al., 2018). *p* < 0.05 was regarded as statistically significant.

### The Gene Expression Omnibus (GEO)

The gene expression microarray of GSE20347 and GSE38129 were downloaded from the Gene Expression Omnibus (GEO, https://www.ncbi.nlm.nih.gov/geo/) of the National Center for Biotechnology Information (NCBI) via the GEO 1010query package (Davis and Meltzer, 2007). Altogether, 17 tumors and matched normal adjacent tissue samples were obtained from GSE20347, while 30 ESCC and 30 normal samples were obtained from GSE38129. The detection platform of the above expression microarrays was GPL571 [HG-U133A_2] Affymetrix Human Genome U133A 2.0 Array.

### GEPIA

GEPIA (http://gepia.cancer-pku.cn/) is a newly generated web server containing RNA sequence expression data of 9,736 tumors and 8,587 normal samples based on TCGA and the GTEx databases (Tang et al., 2017). We used the “Single Gene Analysis” module to perform differential mRNA expression analysis according to pathological stages, survival analysis, and correlation analysis. “Multiple Gene Comparison” module of GEPIA was utilized to assess the multiple gene comparison analysis of the CBX family, using the “ESCA” dataset. The *p* value cutoff was 0.05.

### UALCAN

UALCAN (http://ualcan.path.uab.edu/analysis.html) is a comprehensive database based on level 3 RNA-seq and clinical data of 31 cancer types from The Cancer Genome Atlas (TCGA) and MET500 cohort data (Chandrashekar et al., 2017). Therefore, it can provide information about the relative transcriptional expression of genes in carcinomas compared with normal samples. Furthermore, the database also presents information on the association of transcriptional expression with relative clinicopathological features. In our study, we employed UALCAN to investigate the CBXs mRNA expressions and their relationship with clinicopathological parameters of ESCA. The significant difference of transcriptional expression between groups was evaluated using two-sample Student’s t-test and *p* < 0.01 was regarded as statically significant.

### Kaplan–Meier Plotter

The Kaplan–Meier plotter (http://kmplot.com/analysis/) is a tool that can give information on the effects of 54,000 genes on survival in 21 cancer types (Nagy et al., 2018). We made use of the Kaplan–Meier plotter to evaluate the association between the mRNA expression levels of CBX members with OS of ESCA patients. Information about the number-at-risk cases, median values of mRNA expression levels, the hazard ratio (HR), 95% confidence intervals (CIs), and log-rank *p* value can be accessed at the K-M plotter webpage. A statically significant difference was considered when *p* value was below 0.05.

### cBioPortal

cBioPortal (www.cbioportal.org) is an open-access resource used to visualize and analyze multidimensional cancer genomics datasets (Cerami et al., 2012). Based on the TCGA database, genetic alterations of the CBX gene in patients with ESCA were obtained from cBioPortal. The obtained mRNA expression z-score threshold was 1.8 between the unaltered and altered patients.

### Metascape

Metascape (http://metascape.org) is a free, well-maintained, user-friendly tool for gene annotation and gene list enrichment analysis (Zhou et al., 2019). We used Metascape to perform pathway and process enrichment analysis of the CBX genes and neighboring genes closely related to CBXs alteration via the “Custom Analysis” module, for GO and KEGG enrichment as well as protein-protein interaction analyses.

### TIMER

TIMER (https://cistrome.shinyapps.io/timer) is a comprehensive resource that could provide systematic analyses with the dataset of 10,897 samples among diverse cancers in the TCGA database (Li et al., 2017). CBX family expression scatter plots and their correlation with the abundance of immune cells such as B cells, CD4^+^ T cells, CD8^+^ T cells, neutrophils, macrophages, and dendritic cells in ESCA were assessed using Spearman’s correlation with TCGA_ESCA datasets. The infiltration abundance for each somatic copy number alterations (SCNA) category was compared to the normal by a two-sided Wilcoxon rank-sum test, and statistical significance was identified as *p* < 0.01.

### Clinical Samples

ESCA tissues and the corresponding adjacent normal tissues were collected from patients undergoing surgery at Fujian Union Hospital between April 2020 and April 2021. Patients who received neoadjuvant chemoradiotherapy were excluded. The Ethics Committee of the Fujian Medical University Union Hospital approved for use of all specimens, and all patients provided written informed consent. The tissues were stored in liquid nitrogen until use.

### QRT-PCR Analysis

We quantified the level of CBX family members in tumor tissues (*n* = 17) as well as adjacent normal tissues (*n* = 17) obtained from patients with ESCA, using Quantitative real-time PCR. Total RNA from specimens were isolated using Trizol reagent (Invitrogen). Quantitative RT-PCR was performed with SYBR Green Real-Time Mix (Roche) by a 7,500 Real-time PCR according to the manufacturer’s protocol (Applied Biosystems). PCR amplification was conducted in the following conditions: 95°C for 10 min, 95°C for 15 s (denature), and 60 °C for 1 min (anneal/extend) for 40 cycles, 95 °C for 15 s, and 60 °C for 1 min, and then 95 °C for 15 s (Mel curve). Primers are listed in Supplementary Table S1. The fold change was quantified via 2 − ΔΔCt [ΔΔCt = (ΔCt of genes of interest) − (ΔCt of β-actin)].

### Western Blotting

We extracted protein from tumor tissues (*n* = 12) and adjacent normal tissues (*n* = 12) tissues in radioimmunoprecipitation assay (RIPA) buffer (Beyotime, Shanghai, China). The protein content was determined by the Bicinchoninic acid (BCA) assay kit (Beyotime, Shanghai, China). Proteins were separated by SDS-PAGE and were visualized using the ECL system (Bio-Rad, Hercules, CA, United States). Primary antibodies used in this study were as follows: CBX1 Polyclonal Antibody (Proteintech, 10241-2 -AP), CBX3 Polyclonal Antibody (Proteintech, 11650-2 -AP), CBX7 Polyclonal Antibody (Proteintech, 26278-1-AP), and CBX8 Monoclonal Antibody (Santa Cruz, sc-374332). Monoclonal Anti-GAPDH antibody was purchased from Cell Signaling Technology (Danvers, MA, United States).

### Immunofluorescence Staining

ESCA tumor tissues and adjacent normal tissue were collected after surgery, then were fixed in 10% neutral buffered formalin and embedded in paraffin. Tissue samples were then sectioned at 4um thickness. Sections were deparaffinized, rehydrated, processed for antigen retrieval, blocked, incubated with primary antibody (CBX1:1:500 dilution, CBX3:1:500 dilution, CBX8:1:50 dilution) at 4 °C overnight, followed by incubation corresponding fluorescence-conjugated secondary antibody (1:500 dilution) for 1 h at room temperature. Then slides were incubated with DAPI (BD Biosciences) for 10 min at RT and mounted with ProLong Gold antifade reagent (Invitrogen). Subsequently, the fluorescence images were captured with confocal microscopy.

## Results

### Aberrant Expression and Diagnostic Capability of CBX Family Members in ESCA Patients

**FIGURE 1:**
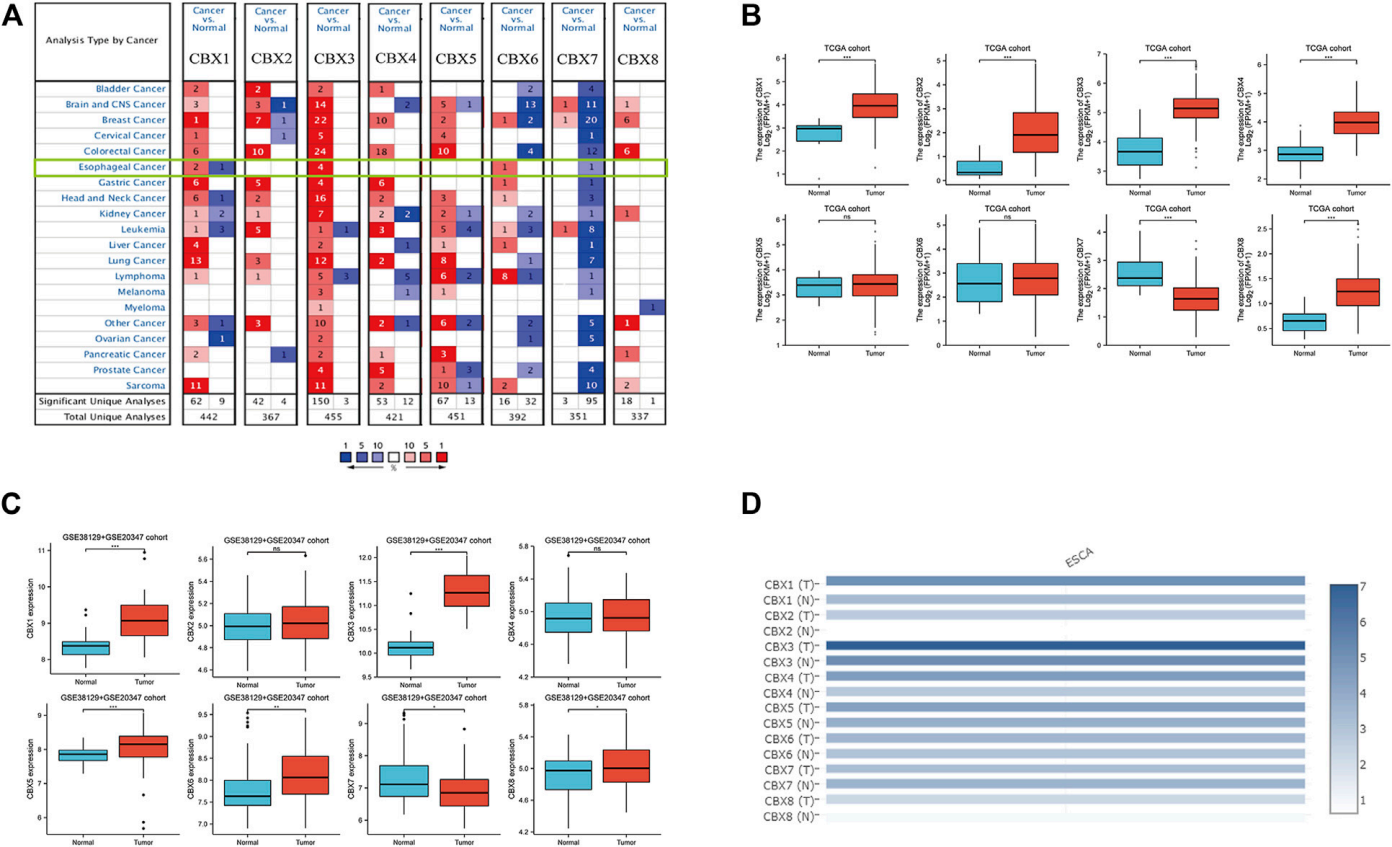
Expression of CBX family members in ESCA and normal. **(A)** mRNA levels of CBX family members in various types of cancer (ONCOMINE). The figure shows the numbers of datasets with statistically significant mRNA overexpression (red) or downregulated expression (blue) of CBXs. Cutoff of *p* value: 0.05, fold change:1.5, gene rank: 10%, data type: mRNA. **(B)** Transcriptional of CBX family members in ESCA and normal (TCGA). **(C)** Expression of CBX family members in ESCA and adjacent normal (GSE38129 + GSE20347 cohort). The *p* value was set at 0.05. **(D)** Relative mRNA expression level of CBXs in ESCA and normal tissues in GEPIA. Color intensity represents the fold change expression of the genes in the tissue. *p < 0.05, **p < 0.01, ***p < 0.001.

The expression of CBX family members were collected using ONCOMINE database (Figure 1A). We first investigated the mRNA transcriptional levels of CBXs in ESCA and normal tissues with ONCOMINE. As shown in Figure 1A and Table 1, the transcriptional levels of CBX1, CBX3, and CBX6 in ESCA were significantly elevated while CBX7 was significantly reduced compared to normal tissues. TCGA ESCA cohort showed that the expression levels of CBX1 (*p* < 0.001), CBX2 (*p* < 0.001), CBX3 (*p* < 0.001), CBX4 (*p* < 0.001), and CBX8 (*p* < 0.001) were higher while the expression level of CBX7 (*p* < 0.001) was lower in ESCA samples compared to paired para-cancerous samples (Figure 1B). Then we evaluated the expression levels of CBXs in ESCA and normal tissues with the GEO database (GSE20347 and GSE38129). The transcriptional levels of CBX1 (*p* < 0.001), CBX3 (*p* < 0.001), CBX5 (*p* < 0.001), CBX6 (*p* = 0.006), and CBX8 (*p* = 0.049) in ESCA were significantly elevated, while CBX7 (*p* = 0.015) was decreased in ESCA tissues compared to normal tissues (Figure 1C). We also compared the relative expression levels of CBXs in ESCA using GEPIA, the results showed that the relative expression of CBX3 was the highest among all the CBX proteins (Figure 1D).

**TABLE 1:** Significant changes of CBX expression in the transcription level between ESCA and normal esophagus tissues (ONCOMINE).

	Types of ESCA vs. normal	Fold change	*p* value	*t*-test	Ref
CBX1	Esophageal squamous cell carcinoma	1.662	5.56E-13	8.101	Su Esophagus 2 (106)
	Esophageal adenocarcinoma	2.780	7.87E-4	3.720	Hao Esophagus (48)
CBX3	Esophageal squamous cell carcinoma	2.188	3.75E-9	15.587	Su Esophagus 2 (106)
	Esophageal squamous cell carcinoma	2.389	3.21E-10	10.596	Hu Esophagus (34)
	Esophageal adenocarcinoma	2.559	1.20E-4	4.887	Kimchi Esophagus (118)
	Barrett’s esophagus	1.629	1.06E-5	5.127	Kim Esophagus (118)
CBX6	Esophageal squamous cell carcinoma	2.101	1.43E-6	6.336	Hu Esophagus (34)
CBX7	Esophageal squamous cell carcinoma	−1.621	5.17 E-8	5.770	Su Esophagus 2 (106)

ESCA: esophagus cancer; CBX: chromobox.

**FIGURE 2:**
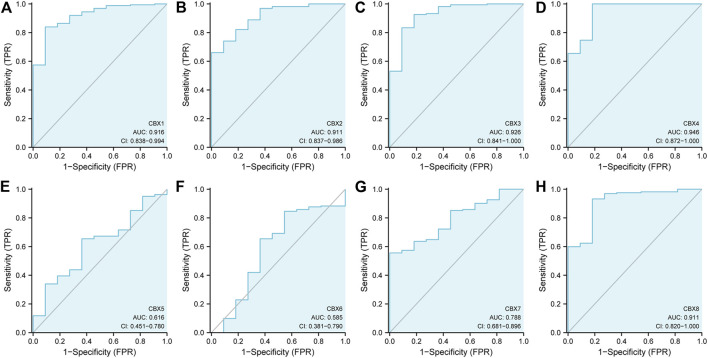
ROC curve analysis of CBXs diagnostic capability in ESCA cancer **(A–H)** (TCGA): Normal versus tumor.

Next, we used the ROC curve of CBX family members to access the diagnostic capability of CBX family members for ESCA by using the TCGA cohort. The results indicated that CBX1, CBX2, CBX3, CBX4 and CBX8 had great diagnostic capability with AUC of 0.916(95% CI: 0.838–0.994),0.911(95% CI: 0.837–0.986),0.926(95% CI: 0.841–1.000),0.946(95% CI: 0.872–1.000), and 0.911 (95% CI: 0.820–1.000), respectively. CBX7 had moderate diagnostic capability with AUC of 0.788 (95% CI: 0.681–0.896). CBX5 and CBX6 had low diagnostic capability with AUC of 0.616 (95% CI: 0.451–0.780) and 0.515 (95% CI: 0.381–0.790) (Figure 2), respectively.

In short, we found CBX1, CBX3, and CBX8 were significantly upregulated in ESCA vs. normal tissues, while CBX7 was downregulated in ESCA. Furthermore, CBX1, CBX2, CBX3, CBX4, and CBX8 had great diagnostic capability to distinguish ESCA from normal tissues.

### Association of the mRNA Expression of CBX Family Members With the Clinicopathological Parameters of ESCA Patients

**FIGURE 3:**
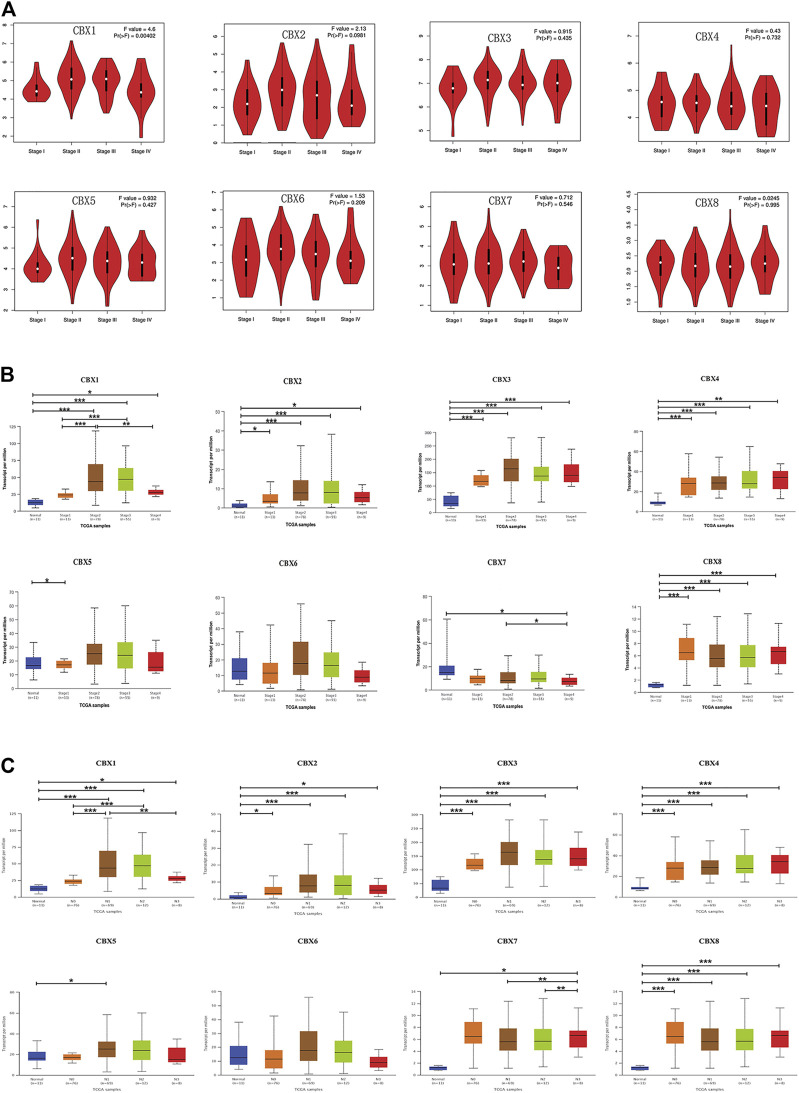
**(A)** Correlation between different expressed CBXs and the pathological stage of ESCA patients in GEPIA. *p < 0.05. **(B)** Relationship between mRNA expression of distinct CBX family members and individual cancer stages of ESCA patients. *p < 0.05, **p < 0.01, ***p < 0.001. **(C)** Association of mRNA expression of distinct CBXs family members with tumor nodal metastasis status of ESCA patients. *p < 0.05, **p < 0.01, ***p < 0.001.

Since the mRNA expression of CBX family members was aberrant in ESCA patients, we next analyzed the relationship between the mRNA expression of different CBX family members and the clinicopathological parameters of ESCA patients. First, we utilized GEPIA to further detect the correlation between the mRNA expression levels of different CBX family members and the pathological stage of ESCA patients. We found a significant correlation between the expression of CBX1 (*p* = 0.004) and the pathological stage (Figure 3A). CBX1 expression was increased in the advanced stage (stage II/III) as compared with those in the early tumor stage (stage I). These data suggest that CBX1 plays a significant role in the tumorigenesis and progression of ESCA.

We also analyzed the relationship between the mRNA expression of different CBX family members and the cancer stages, tumor grades, tumor nodal metastasis status, and TP53 mutation status of ESCA patients by UALCAN (http://ualcan.path.uab.edu). As shown in Figure 3B, mRNA expressions of CBX1, CBX2, CBX3, CBX4, and CBX8 were upregulated in ESCA as compared with normal. The expression of CBX1 tended to be higher as the tumor stage increased, which was consistent with the previous findings in the GEPIA database. Nevertheless, the mRNA expression of CBX7 was the highest in normal tissues, and as the cancer stage increased, the mRNA expression of CBX7 tended to decrease. CBX6 mRNA expression had no significant relationship with tumor stages. Similarly, mRNA expressions of CBX1, CBX2, CBX3, CBX4, and CBX8 were significantly related to patients’ tumor nodal metastasis status. Patients who were in more advanced tumor nodal metastasis status tended to express higher mRNA of CBX1. However, normal tissues had the highest mRNA expression of CBX7, and the mRNA expression of CBX7 tended to be lower as tumor nodal metastasis status increased (Figure 3C). We then assessed the relationship between the different expressions of CBX family members and the tumor grades of ESCA patients. As shown in Supplementary Figure S1A, mRNA expressions of CBX1, CBX2, CBX3, CBX4, and CBX8 were significantly associated with tumor grades. The mRNA expression of CBX1, CBX2, CBX3, CBX4, and CBX8 tended to be elevated as the tumor grade increased. However, the mRNA expression of CBX7 tended to be lower in grade 4 compared with grade 3. TP53 mutation has been recognized as the most common event and frequently occurs in ESCA (Yang et al., 2020). Interestingly, as shown in Supplementary Figure S1B, CBX family members’ expressions were associated with TP53 mutation status in ESCA patients. CBX1, CBX2, CBX3, and CBX8 were upregulated in ESCA patients with TP53 mutation compared with normal tissues and TP53 non-mutation patients.

Taken together, these data suggested that the mRNA overexpression of CBX1, CBX3, and CBX8 were significantly related to tumor grades and patients’ tumor nodal metastasis status. CBX1, CBX2, CBX3, and CBX8 were significantly related to TP53 mutation in ESCA patients.

### The Prognostic Value of CBX Family Members in ESCA Patients

To evaluate the value of differentially expressed CBX family members in the progression of ESCA, we assessed the correlation between differentially expressed CBX family members and clinical outcomes using GEPIA. Disease-free survival curves (DFS) were presented in Supplementary Figure S2A. ESCA patients with lower transcriptional levels of CBX1 (HR = 1.6, *p* = 0.044) were significantly associated with longer DFS. The value of differentially expressed CBX in the overall survival of ESCA patients was also evaluated (Supplementary Figure S2B). CBX family members did not seem to have a significant effect on Overall Survival (OS).

We also analyzed the prognostic values of CBX family members in patients with ESCA using the TCGA database. Also, the result suggested that ESCA patients with higher CBX3 ((HR = 1.78, *p* = 0.026)) and CBX4 (HR = 1.94, *p* = 0.011) levels had shorter OS (Supplementary Figure S3A). Increased CBX4 (HR = 2.07, *p* = 0.017) and CBX5 (HR = 1.94, *p* = 0.03) mRNA levels were remarkably associated with shorter DFS (Supplementary Figure S3B). Moreover, increased CBX 1 (HR = 1.71, *p* = 0.045) mRNA level was significantly correlated with shorter Progress Free Interval (PFI) (Supplementary Figure S3C).

**FIGURE 4:**
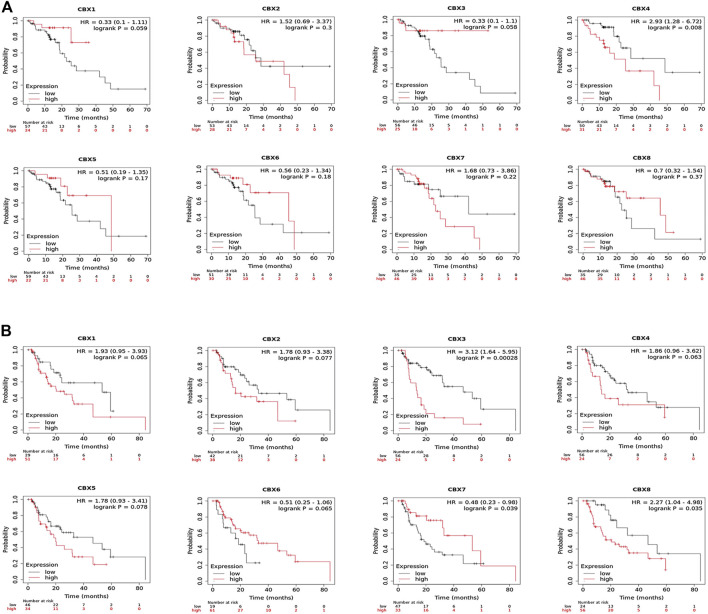
**(A)** Prognostic value of CBXs in esophageal squamous cell carcinoma (ESCC) patients in the overall survival curve (Kaplan–Meier Plotter). **(B)** Prognostic value of CBX family members in esophageal adenocarcinoma (EAC) patients in the overall survival curve (Kaplan–Meier Plotter).

Likewise, we used the Kaplan–Meier plotter to analyze the prognostic values of CBX family members in patients with ESCA subgroup analysis (Figure 4). Esophageal squamous cell carcinoma patients with the higher transcriptional level of CBX4 (HR = 2.93, *p* = 0.008) were closely associated with shorter OS (Figure Figure4A). The overexpression of CBX3 (HR = 3.12, *p* = 0.00028) and CBX8 (HR = 2.27, *p* = 0.035) mRNAs in esophageal adenocarcinoma patients were significantly correlated with shorter OS, whereas the overexpression of CBX7 mRNA (HR = 0.48, *p* = 0.039) was significantly correlated with longer OS (Figure 4B).

Overall, these data demonstrated that increased CBX1/3/4/5/8 and decreased CBX7 were significantly associated with unfavorable clinical outcomes in ESCA patients.

### Genetic Alteration, Expression, and Interaction Analyses of CBX Family Members in ESCA Patients

**FIGURE 5:**
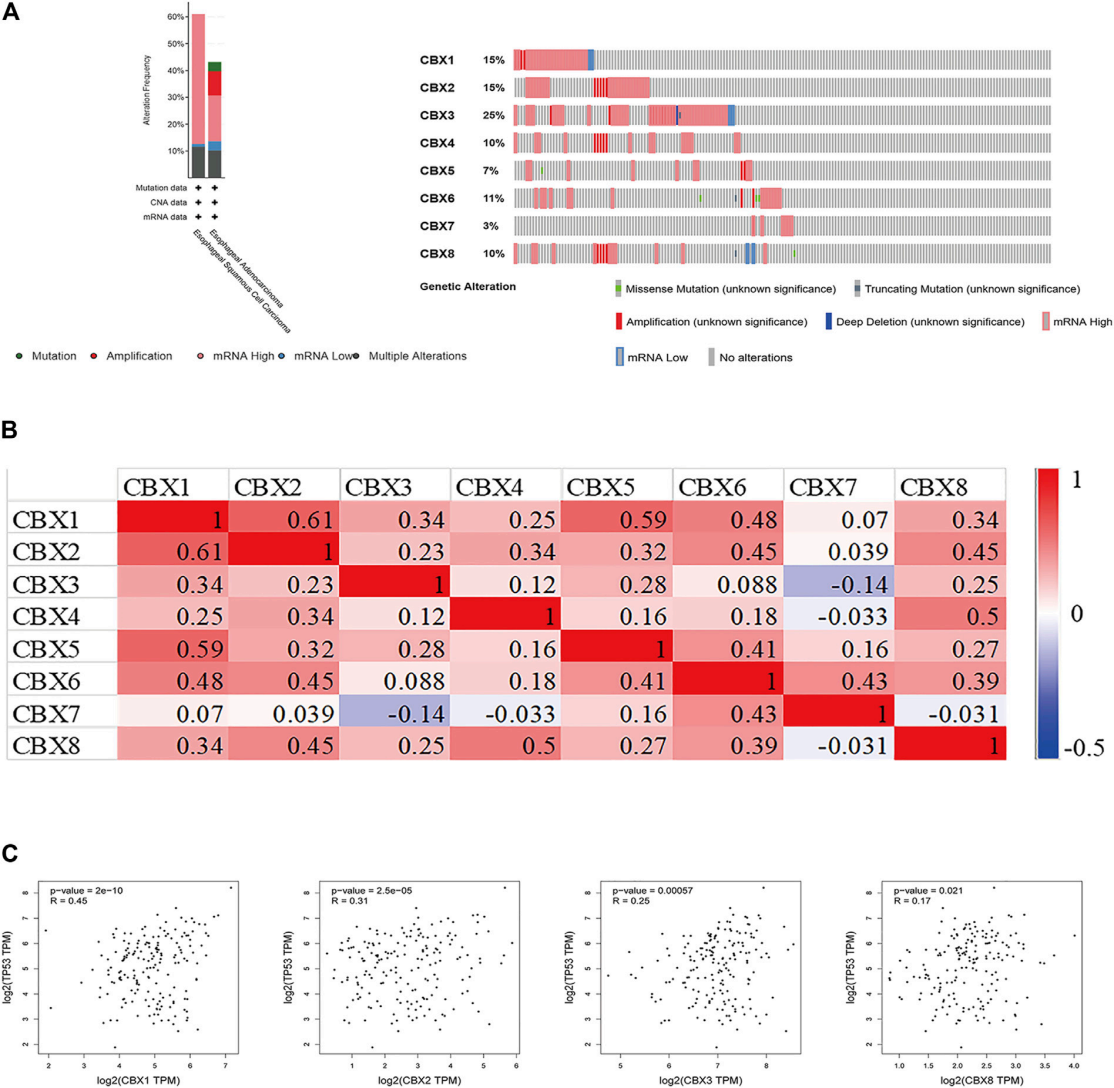
**(A)** Summary of alterations in different expressed CBXs in ESCA. CBXs were altered in 96 samples of 183 patients with ESCA, accounting for 52% (cBioPortal). **(B)** Correlation between different CBXs in ESCA(GEPIA). **(C)** Correlation between CBX family members and TP53 expression (GEPIA). The *p* value was set at 0.05 (GEPIA).

Using cBioPortal, we investigated the genetic alterations of CBX family members and discovered a high alteration frequency (52%) in ESCA patients. CBX3, CBX1, and CBX2 ranked as the top three genes with genetic alterations, which altered in 25, 15, and 15% of the queried ESCA samples, respectively. The primary alteration type in these samples was the enhanced mRNA expression (Figure 5A). Besides, we calculated the correlations of CBXs with each other in GEPIA online tool for correlation analysis in ESCA patients and included Pearson’s correlation. The results showed that there were co-expression associations between the following CBX proteins: CBX1 positively with CBX2/3/4/5/6/8, CBX2 positively with CBX1/3/4/5/6/8, CBX3 positively with CBX1/2/5/8, CBX4 positively with CBX1/2/5/6/8, CBX5 positively with CBX1/2/3/4/6/7/8, CBX6 positively with CBX1/2/4/5/7/8, CBX7 positively with CBX5/6, and CBX8 positively with CBX1/2/3/4/5/6 (Figure 5B). Furthermore, CBX1/2/3/8 were positively co-expressed with TP53 (Figure 5C).

### Enrichment of CBX Family Members and Their 50 Frequently Altered Neighbor Genes’ Ontology in ESCA Patients

**FIGURE 6:**
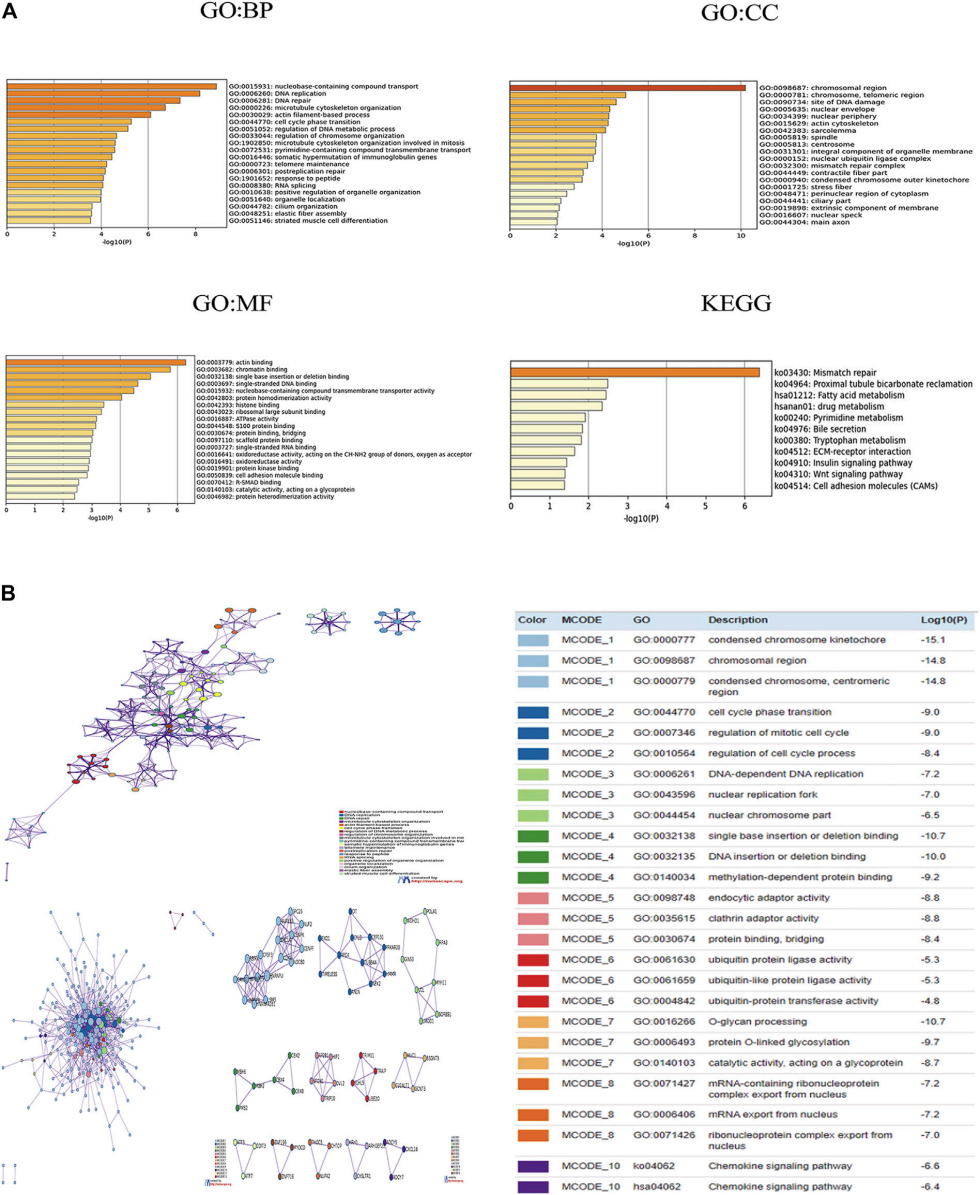
**(A)** Gene Ontology (GO) analysis and Kyoto Encyclopedia of Genes and Genome (KEGG) analysis of CBX genes and similar genes. **(B)** Network of enriched terms of CBX genes and similar genes; Protein-protein interaction (PPI) enrichment analysis of these genes and MCODE (Molecular Complex Detection) components identified in the gene lists. Analyses were conducted in Metascape.

Using cBioPortal, we found 50 genes were most associated with each CBX family member. Some genes were positively associated with CBX family members, whereas others were negatively associated with the proteins. We used Metascape for Gene Ontology (GO) analysis of each CBX protein for biological processes, cellular components, and molecular functions. Moreover, functions of CBX family members and their 50 frequently altered neighbor genes were analyzed by Kyoto Encyclopedia of Genes and Genome (KEGG) and protein-protein interaction (PPI) enrichment analyses in Metascape. As shown in Figure 6A, biological processes such as GO: 0015931 (nucleobase-containing compound transport), GO:0006260 (DNA replication), GO:0006281 (DNA repair), GO: 0000226 (microtubule cytoskeleton organization), and GO: 0030029 (actin filament-based process) were prominently regulated by the CBX family members mutations in ESCA. Cellular components, including GO: 0098687 (chromosomal region), GO: 0000781 (chromosome, telomeric region), GO: 0090734 (site of DNA damage), GO: 0005635 (nuclear envelope) and GO: 0034399 (nuclear periphery) were remarkably associated with the CBX family members alterations. Besides, CBX family members mutations also significantly affected the molecular functions, such as GO: 0003779 (actin-binding), GO: 0003682 (chromatin binding), GO: 0032138 (single base insertion or deletion binding), GO: 0003697 (single-stranded DNA binding), and GO: 0015932 (nucleobase-containing compound transmembrane transporter activity).

In KEGG analysis, 11 pathways including ko:03430 (mismatch repair), ko:04512 (ECM-receptor interaction), ko:04910(Insulin signaling pathway), ko:04310 (Wnt signaling pathway), and ko:04514(Cell adhesion molecules (CAMs) were associated with the functions of CBX family members mutations in ESCA (Figure 6A). Figure 6B presented the PPI network associated with the genes; it was mainly related to cell cycle, gene mutation, DNA replication, and epigenetics.

### Immune Cell Infiltration Analysis of CBX Family Members in ESCA

Accumulating evidence indicated that immune cell infiltrations are closely related to tumor progression and clinical outcome. In this study, we also explored the correlation between CBX family members and immune cell infiltration in ESCA by utilizing the TIMER database (Supplementary Figure S4). There was a positive correlation between CBX1 expression and the infiltration of macrophages (Cor = 0.171, *p* = 2.20e-02), and a negative correlation between CBX1 expression and the infiltration of neutrophils (Cor = −0.15, *p* = 4.45e-02). CBX2 expression was negatively associated with the infiltration of CD8^+^ T cells (Cor = −0.184, *p* = 1.35e-02). CBX3 expression was negatively associated with the infiltration of dendritic cells (Cor = −0.249, *p* = 7.38e-04). Similarly, the expression of CBX5 was positively associated with the infiltration of macrophages (Cor = 0.199, *p* = 7.27e-03). There was a negative correlation between CBX6 expression and the infiltration of CD8^+^ T cells (Cor = -0.167, *p* = 2.51e-02), and a positive correlation between CBX6 expression and the infiltration of macrophages (Cor = 0.263, *p* = 3.62e-04). There was a positive correlation between CBX7 expression and the infiltration of B cells (Cor = 0.254, *p* = 5.87e-04), CD4^+^ T cells (Cor = 0.183, *p* = 1.43e-02) and macrophages (Cor = 0.283, *p* = 1.20e-04). The expression of CBX8 was positively associated with the infiltration of B cells (Cor = 0.222, *p* = 2.82e-03), and negatively associated with the infiltration of neutrophils (Cor = −0.164, *p* = 2.78e-02), and dendritic cells (Cor = −0.375, *p* = 2.09e-07).

**FIGURE 7:**
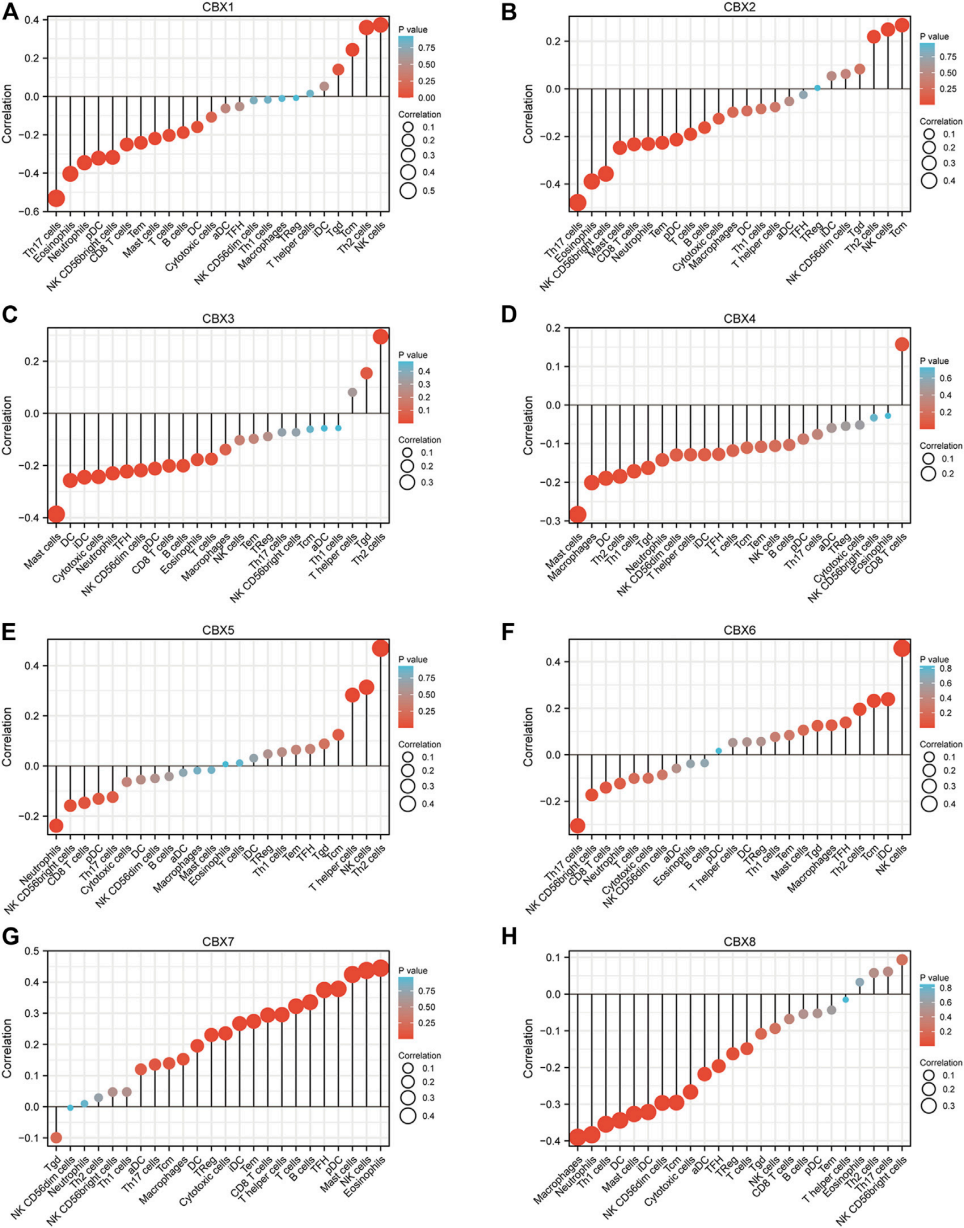
Correlations between differentially expressed CBX family members and immune cell infiltrations (TCGA). Correlations between the abundance of immune cells and the expression of CBX1-8 **(A–H)**.

TCGA ESCA project in level 3 HTSeq-FPKM format RNAseq data and clinical data showed that CBX1, CBX2, CBX3, CBX4, and CBX8 were negatively related to the infiltration of most immune cells, while CBX7 was positively associated with the infiltration of most immune cells (Figure 7). We also analyzed the relationship between CBX1/3/7/8 expression levels and immune cell infiltration in ESCA. Patients in the high CBX1 expression group (*n* = 173, data from TCGA database) presented a decrease in the numbers of infiltrating T cells, B cells, CD8 T cells, neutrophils, and Th17 cells (Supplementary Figure S5A). The group with higher expression of CBX3 showed a decrease in immune cell infiltration, including T cells, B cells, cytotoxic cells, CD8 T cells, neutrophils, and Th17 cells (Supplementary Figure S5B). The higher CBX7 expression group presented higher infiltration of immune which can kill tumor cells, including T cells, B cells, Cytotoxic cells, CD8 T cells, NK cells, iDC cells, Treg cells, and Th17 cells (Supplementary Figure S5C). Patients in the high CBX1 expression group indicated a decrease in the numbers of infiltrating T cells, cytotoxic cells, neutrophils, iDC cells, macrophages cells, and Treg cells (Supplementary Figure S5D).

These indicated that CBX family members may interact with immune cell infiltration, and then influence the outcomes of ESCA patients.

### Association of CBX Family Members With PRC2 in ESCA Patients

Aberrant epigenetic regulation has been reported to promote the pathogenesis and progression of ESCA. The methylation of lysine 27 on histone H3 (H3K27me3) is a chromatin marker associated with nucleosome condensation and silencing of gene expression (Adema and Colla, 2022). PCR1 and PCR2 played critical roles in establishing and maintaining the H3K27me3 mark. PRC2 comprised three core components (EZH2, SUZ12, and EED). EZH2 has hmtase activity, which is maintained by the presence of SUZ12 and EED (Erokhin et al., 2021). We used TCGA databases to analyze the expression levels of PRC2 components in ESCA. As shown in Supplementary Figure S6A, the transcriptional levels of EZH2, SUZ12, and EED were significantly elevated in ESCA tissues compared to normal tissues. We further used TCGA databases to analyze the association of CBX family members with PRC2 components in ESCA patients. The results showed that except CBX7, other CBX family members were positively correlated with PRC2 components, among which CBX1/3/8 had the highest correlation. (Supplementary Figure S6).

### Validation of CBX Family Members in Clinical Samples

**FIGURE 8:**
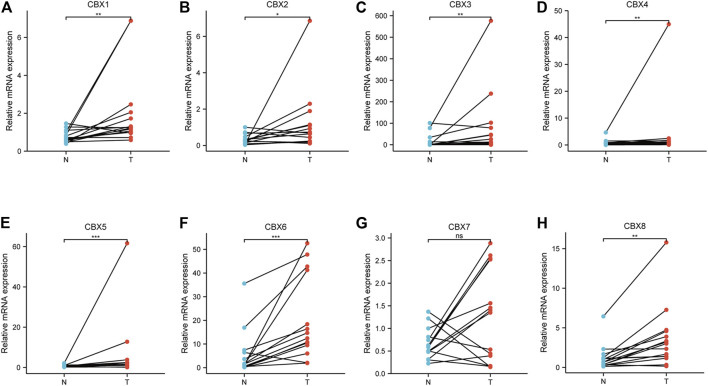
Real-time qPCR validation of CBX family members in 17 ESCA and normal esophageal tissues **(A–H)**. *p < 0.05, **p < 0.01, ***p < 0.001 analysis by paired t-test.

To validate the finding in the mentioned databases and further reveal which CBX members play a crucial role in the progression of ESCA, we used real-time PCR to detect all the mRNA expression of CBX family members using clinical samples. The analysis results showed that CBX1, CBX2, CBX3, CBX4, CBX5, CBX6, and CBX8 were significantly higher in ESCA tissues compared to normal esophageal tissues, which is consistent with the results of bioinformatics analysis mentioned previously (Figures 8A–F,H). However, CBX7 was downregulated in tumor tissues in half of collected ESCA patients, although there was no difference in all patients (Figure 8G).

**FIGURE 9:**
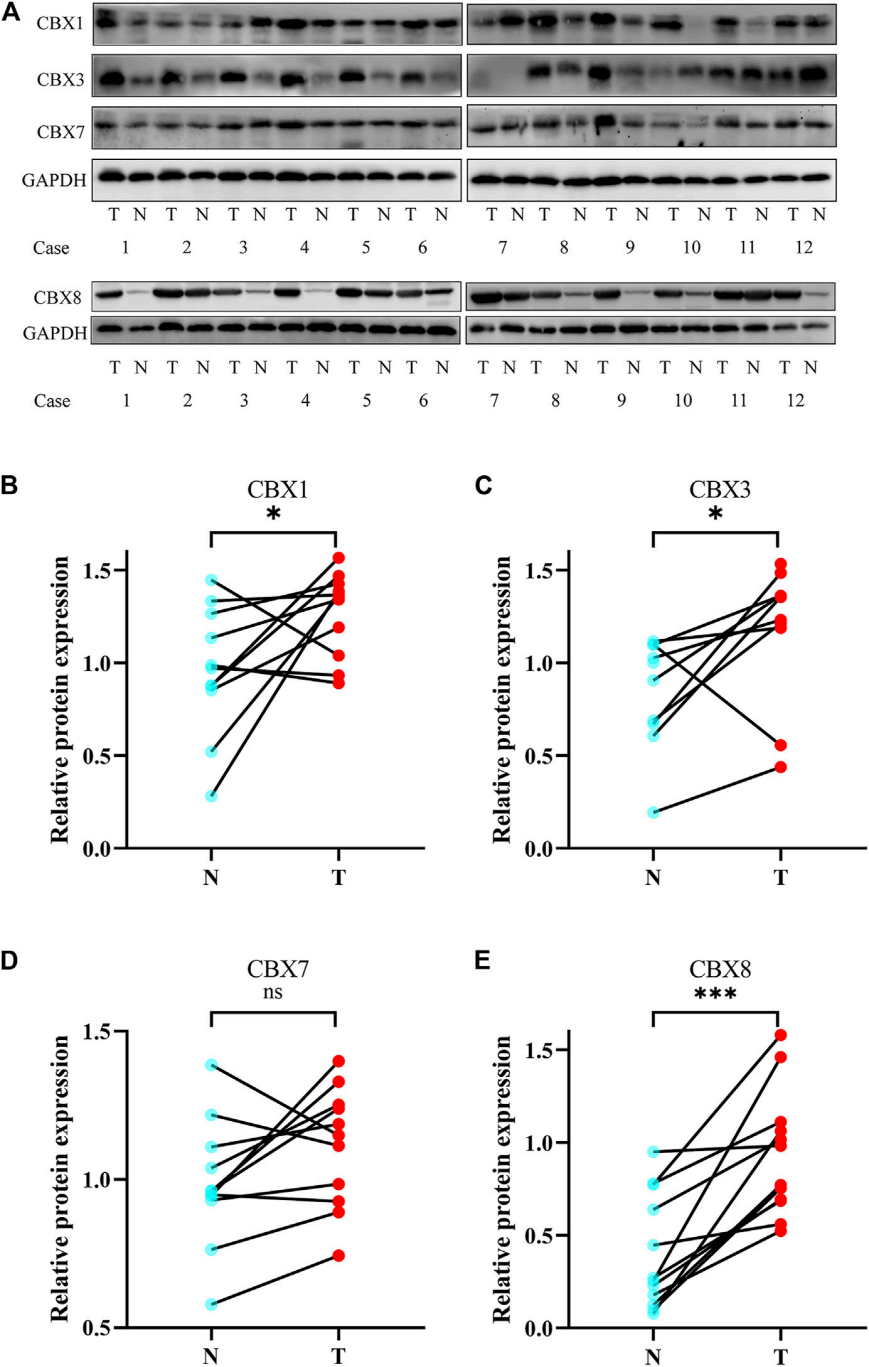
Protein expression levels of CBX1, CBX3, CBX7, and CBX8 in 12 ESCA tissues and adjacent normal tissues (western blot). **(A)** CBX1, CBX3, and CBX8 were upregulated in ESCA tissues compared with adjacent normal tissues, while CBX7 was expressed at similar levels in ESCA and normal esophageal tissues. **(B–E)** Statistical results of western blotting of CBX1, CBX3, CBX7, and CBX8 in ESCA tissues and normal tissues. (*p < 0.05, analysis by paired t-test.)

**FIGURE 10:**
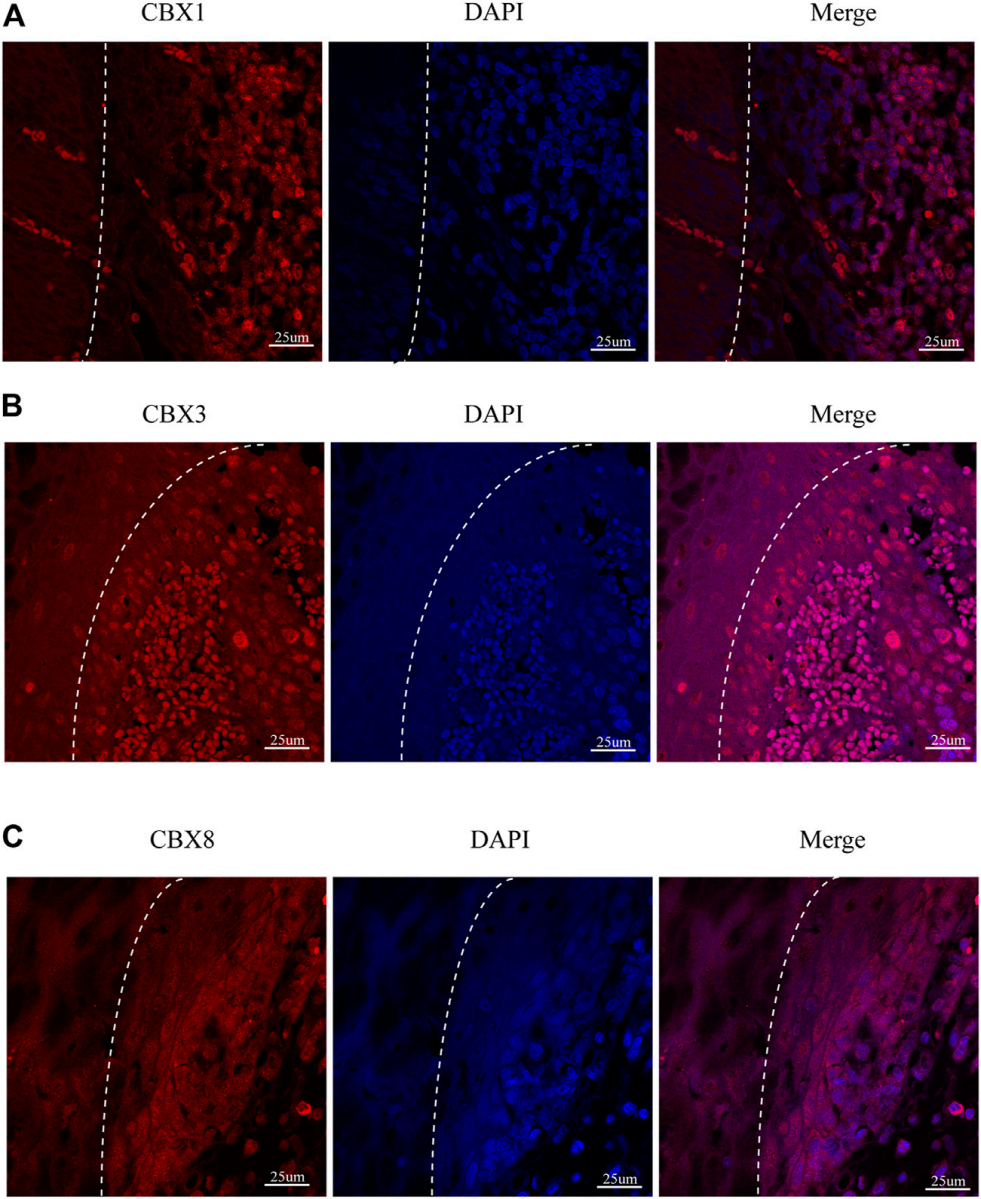
Fluorescence images of paraffin-embedded sections of human ESCA and adjacent tissues with confocal microscopy. **(A)** CBX1 was significantly higher in ESCA tissues than that in precancerous tissues. **(B)** CBX3 was significantly higher in ESCA tissues than that in precancerous tissues. **(C)** CBX8 was significantly higher in ESCA tissues than that in precancerous tissues. The white dashed lines separate the precancerous tissues (on the left side of the figure) from the ESCA tissues (on the right side of the figure). Scale bars = 25 um. (Magnification 63✕/1.40 oil).

We also investigated the protein expression of CBXs in ESCA tissues using western blotting and immunofluorescence staining. Western blotting results showed that the protein expression of CBX1, CBX3, and CBX8 were much higher in ESCA tissues than precancerous tissues (Figure 9). Immunofluorescence staining showed that the expression of CBX1, CBX3, and CBX8 were significantly higher in ESCA than adjacent normal tissues, which corroborated the results of western blot assay (Figure 10).

## Discussion

ESCA is one of the most common and aggressive malignancies worldwide, with dismal clinical outcomes. It is universally acknowledged that ESCA evolution is a long-term, multistep process that begins from basal cell hyperplasia, low-grade dysplasia, high-grade dysplasia, carcinoma *in situ* to invasive carcinoma, and metastasis (Wang et al., 2005; Wei et al., 2015). Over the past couple of decades, genomic and epigenomic factors have been widely illustrated, which take part in the transformation of esophageal squamous precancerous lesions into ESCA (Lin et al., 2018). In addition to cancer genetics, abnormal epigenetic regulation including abnormal DNA methylation, aberrant histone modifications, and alterations of various non-coding RNAs have also been found to take an important part in driving the pathogenesis and progression of ESCA(Cao et al., 2020). Being important components of epigenetic regulation complexes, CBX family members affect the carcinogenesis and development of various cancers, including ESCA, liver cancer, and breast cancer. Despite some members of CBXs proteins having been confirmed to be implicated in ESCA, diverse roles of eight CBX family members in ESCA remain to be fully elucidated. In this study, we used various large databases to explore the role of CBX family members in ESCA with five aspects: expression pattern, clinicopathological parameters, prognostic value, genetic mutation, and immune cell infiltration.

Recently, studies from Li et al. discovered that high CBX1 expression was connected to aggressive types of breast cancers (TNBC phenotype), and the patients were inclined to have P53 mutations and lymph node metastasis (Li X et al., 2020). Prognosis analysis showed that high CBX1 was related to worse distant metastasis-free survival in breast cancer patients (Li X et al., 2020). Noticeably higher mRNA and protein expressions of CBX1 were discovered in hepatocellular carcinoma tissues compared to normal tissues significantly linked with shorter OS (Ning et al., 2018). CBX1 overexpression promoted HCC cell growth and migration by activating the Wnt/β-Catenin signaling pathway, whereas inhibition of CBX1 or knockdown of β-Catenin markedly decreased CBX1-mediated cell proliferation (Yang et al., 2018). The present study found that the mRNA expression of CBX1 in ESCA was higher than that in normal tissues in databases (TCGA and GEO cohort), which were verified by RT-PCR, western blot, and immunofluorescence in our clinical samples. ROC curve analyses showed that CBX1 had great diagnostic capability to distinguish ESCA from normal. Also, we found that overexpression of CBX1 in ESCA is significantly correlated with clinical tumor stage, tumor nodal metastasis status, tumor grade, and TP53 mutation status. Furthermore, the overexpression of CBX1 mRNA was markedly related to shorter DFS and PFI, indicating CBX1 took part in the tumorigenesis of ESCA. These results implied that CBX1 is a tumor promoter and biomarker for ESCA patients.

Growing evidence demonstrated that CBX3 deserves attention in the tumorigenesis and treatment of human malignancies. In patients with non-small cell lung cancer (NSCLC), elevated CBX3 expression is associated with poor survival (Chang et al., 2018). Dramatic upregulation of CBX3 had been found in colorectal cancer (CRC) tissues, which are related to unfavorable DFS(Li Q et al., 2020). CBX3 has been identified as a positive regulator of aerobic glycolysis and promotes growth by suppressing fructose-1,6-bisphosphatase 1 in pancreatic cancer (Chen et al., 2018). In glioma patients, CBX3 is dramatically upregulated in tumor tissues and cells, correlated with unfavorable prognosis, and it could regulate the proliferation of glioma U87 cells through CDKNIA (S. Zhao et al., 2019). Moreover, in patients with HCC and breast cancers, CBX3 overexpression promotes the proliferation of tumor cells and is associated with poor prognosis (Liang et al., 2017; Zhong et al., 2019). Herein, consistent with these previous studies, we indicated that CBX3 mRNA was upregulated in ESCA (both TCGA cohort and GEO cohort) and this expression was significantly related to tumor grades, tumor nodal metastasis status, and TP53 mutation status. ROC curve analyses indicated that CBX3 had great diagnostic capability in ESCA. In addition, overexpression of CBX3 was dramatically associated with shorter OS of ESCA patients. Therefore, we postulate that CBX3 takes part in the occurrence and progression of ESCA and might be a prognosis biomarker in ESCA.

Paradoxical roles of CBX7 had been shown in different malignant carcinomas (Pallante et al., 2015). For example, CBX7 has been recently demonstrated to be overexpressed in ovarian cancer and reduced overall survival rates compared with patients not expressing CBX7 (Shinjo et al., 2014). Furthermore, studies have shown that CBX7 could combine with E-box to inhibit tumor proliferation and migration via suppressing TWIST1 function (Li J et al., 2020). Consistently with an oncogenic role, CBX7 controls the lifespan of various human primary cells, also immortalizing mouse fibroblasts through the regulation of the Ink4a/Arf locus (Gil et al., 2004). Conversely, CBX7 plays as a tumor suppressor and is negatively correlated with cancer aggressiveness. CBX7 was downregulated in gastric cancer tissues compared to normal tissues, and this downregulation of CBX7 was closely related to poor OS (Ma et al., 2020). CBX7 reduces the emergence of breast adenocarcinoma by inhibiting the Wnt/β-catenin pathway via upregulation of the Wnt antagonist DKK-1 expression (Kim et al., 2015). In our study, CBX7 was downregulated in ESCA and had moderate diagnostic capability in ESCA. Further studies showed that CBX7 was negatively correlated with tumor stage, tumor grade, and tumor nodal metastasis status. Besides, the downregulation of CBX7 mRNA was markedly correlated with poor OS in ESCA. These findings indicated that CBX7 functions as an anti-cancer effect in ESCA.

Upregulation of CBX8 had been revealed in HCC tissues and indicated a worse prognosis in patients (Gao et al., 2015). *In vitro* study had shown that the high expression of CBX8 facilitated tumor proliferation and metastasis by stimulating the AKT/β-catenin pathway (Zhang et al., 2018). Likewise, remarkably higher expression of CBX8 was also observed in HCC tissues, which was significantly linked to cancer stages and tumor grades. Furthermore, high expression of CBX8 was dramatically correlated with shorter OS in patients with liver cancer (Ning et al., 2018). CBX8 performs a conflicting role in ESCA: it promotes cell proliferation but inhibits cell migration, invasion, and metastasis (Wang et al., 2017). In this study, CBX8 was upregulated in ESCA, and it was significantly correlated with clinical tumor grade, tumor nodal metastasis status, and TP53 mutation status. CBX8 showed great diagnostic capability in ESCA. Furthermore, the high expression of CBX8 in Esophageal Adenocarcinoma patients was significantly related to poor OS, indicating CBX8 involved in the tumorigenesis of ESCA.

Additionally, the genetic analysis demonstrating high genetic alterations of CBXs were found in ESCA patients, and the most alteration was the high expression of mRNA. There was a mutually cooccurring connection between different CBXs, indicating that CBXs take an antagonistic or synergistic role in the tumorigenesis of ESCA. Then we found 50 genes most associated with each CBX gene by using cBioPortal. These genes were further annotated based on GO enrichment analysis and KEGG pathway enrichment analysis. The results indicated that the roles of these genes are found to be primarily associated with DNA replication and DNA repair, signaling pathways that involved Mismatch repair (MMR), and Wnt signaling pathways. DNA MMR genes play critical roles in retaining genome stability. It is widely known that TP53 is a tumor suppressor gene, implicated in the regulation of cell growth, apoptosis, cell cycle, differentiation, and senescence in ESCA. TP53 gene mutations and protein accumulations are early and frequent events in ESCA (Shimada, 2018). Patients with ESCA and TP53 gene mutations were correlated with poor overall survival compared with patients without TP53 mutations (Fisher et al., 2017). In our study, we found CBX1/2/3/8 was positively co-expressed with TP53. CBX1/2/3/8 were upregulated in ESCA patients with TP53 mutation compared with normal tissues and TP53 non-mutation patients. CBX family members may promote ESCA development through the P53 pathway.

In recent years, the tumor microenvironment (TME) is becoming increasingly relevant in cancer research (Xu et al., 2021). Immune cells in TME may play tumor-promoting and suppressive roles, thereby influencing the clinical outcome (Yang et al., 2019). The CBX family members have been reported to contribute to the infiltration of immune cells in various cancers (Li Q et al., 2020; Zhou et al., 2021). In our study, we showed that the CBX family members’ expression could be significantly related to the infiltration of immune cells in ESCA, indicating that CBXs might also affect the immune status. In particular, CBX1and CBX3 were negatively related to the infiltration of CD8^+^T cells. CD8^+^T cells are cytotoxic T lymphocytes which are generally considered as the main component of anti-tumor immunity (Mahmoudi et al., 2021). In various cancers, increased CD8^+^ T cell infiltrations in the tumor mass are associated with improved patient survival (Zhao et al., 2018). In addition, in this study, we found CBX7 was positively associated with the infiltration of most immune cells, including T cells, B cells, cytotoxic cells, CD8^+^T cells, NK cells, iDC cells, and Treg cells. These results indicated that CBX7 may play a key role in influencing the immune status of ESCA.

Polycomb group (PcG) proteins including PRC1 and PRC2, are essential epigenetic regulators that maintain transcriptional repression (Vizán et al., 2020). PRC2 consists of three core components [Enhancer of zeste homolog 2 (EZH2), suppressor of zeste 12 (SUZ12), and embryonic ectoderm development (EED)], which medicated histone methyltransferase activity (Zheng et al., 2021). Overexpression of EZH2 in cancer cells results in transcriptional repression through increased H3K27me3 activity. Enzymatic action by EZH2 at target genes requires the binding of SUZ12 and EED. We analyzed the expression levels of PRC2 components and found EZH2, SUZ12, and EED were upregulated in ESCA. Furthermore, CBX family members (especially CBX1/3/8) were positively correlated with PRC2 components. Hence, we believe that the CBX family, as members of PRC1, promote tumorigenesis by interacting with members of PRC2 through establishing and maintaining the H3K27me3 mark.

## Conclusion

In conclusion, we systematically investigated the various expression and prognostic values of CBX family member genes in ESCA using bioinformatics analyses, and we verified these results in tissue samples. Our findings indicated that CBX1/3/8 are tumor promoters, while CBX7 serves as a tumor suppressor in ESCA. Although molecular mechanism studies are needed to validate our findings, our work provides new insights to improve the accuracy of prognosis and precision therapy for ESCA patients.

## Data Availability

The datasets presented in this study can be found in online repositories. The names of the repository/repositories and accession number(s) can be found in the article/Supplementary Material.
