# Supplementary figures and images for: Identification and Validation of Chromobox Family Members as Potential Prognostic Biomarkers and Therapeutic Targets for Human Esophageal Cancer
Source: Front Genet. 2022 Apr 6;13:851390. doi: 10.3389/fgene.2022.851390 (PMC9019303; doi:10.3389/fgene.2022.851390)

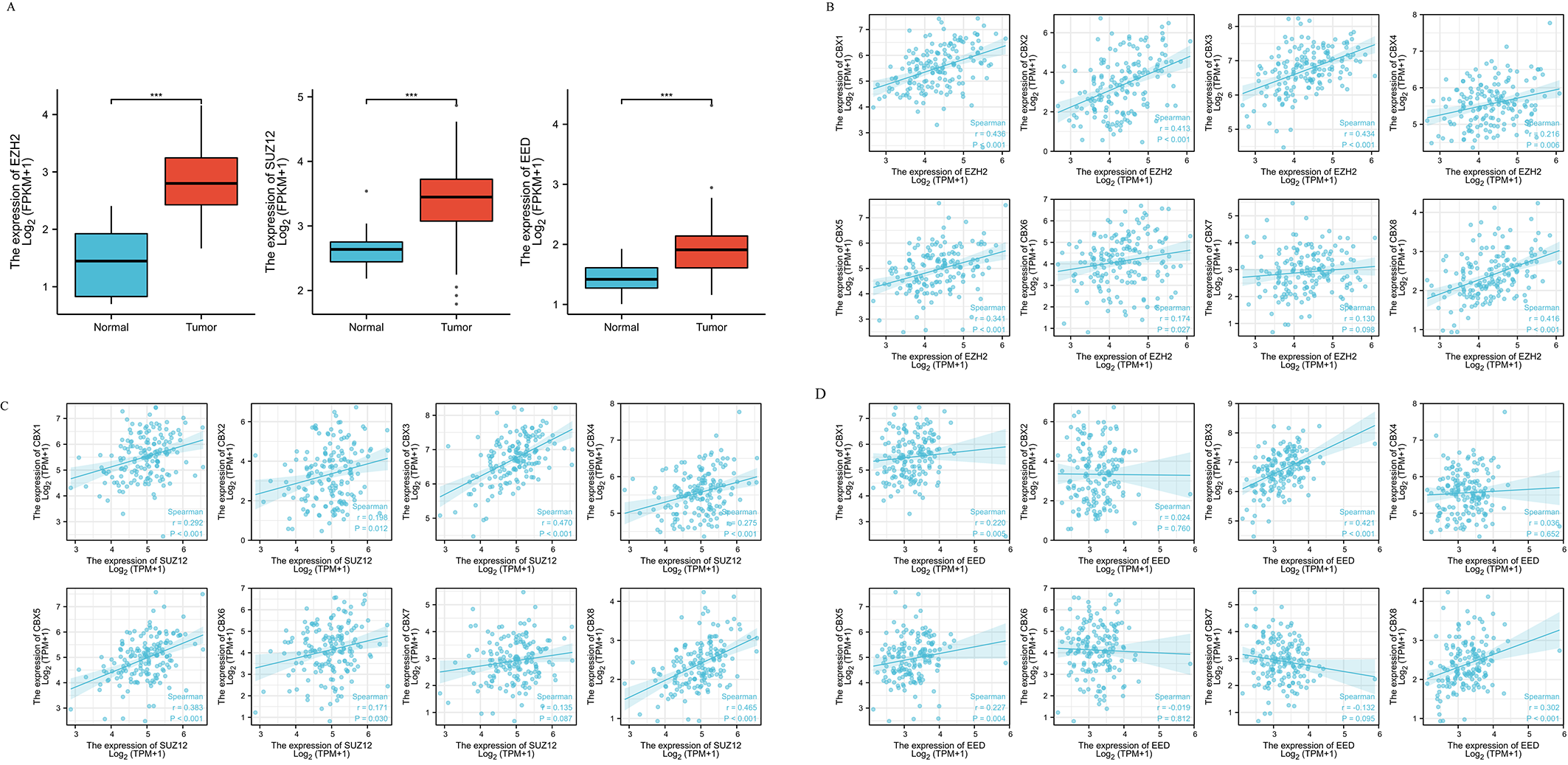

Supplement: Supplementary file 1 [file Image6.TIF]

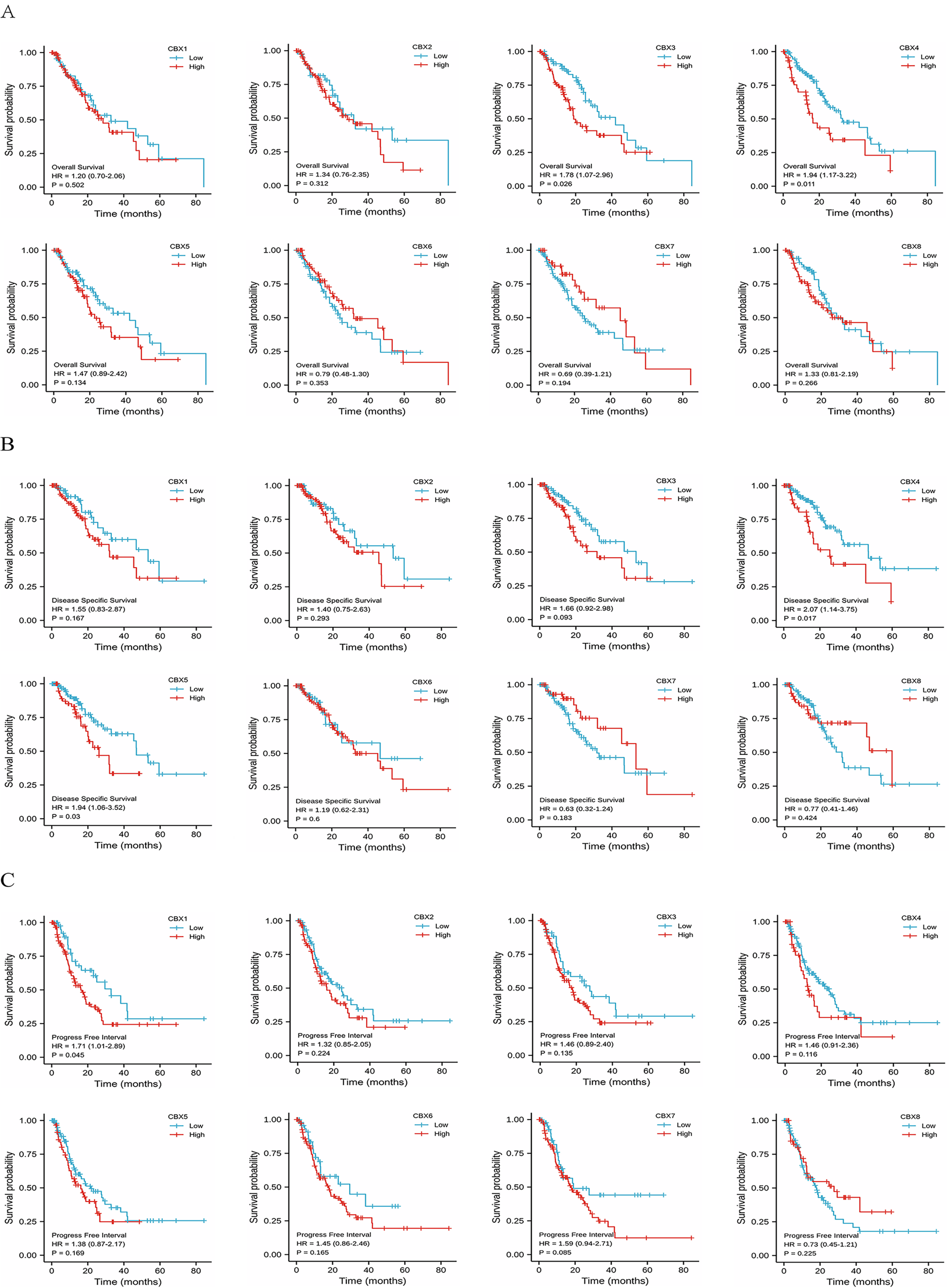

Supplement: Supplementary file 2 [file Image3.TIF]

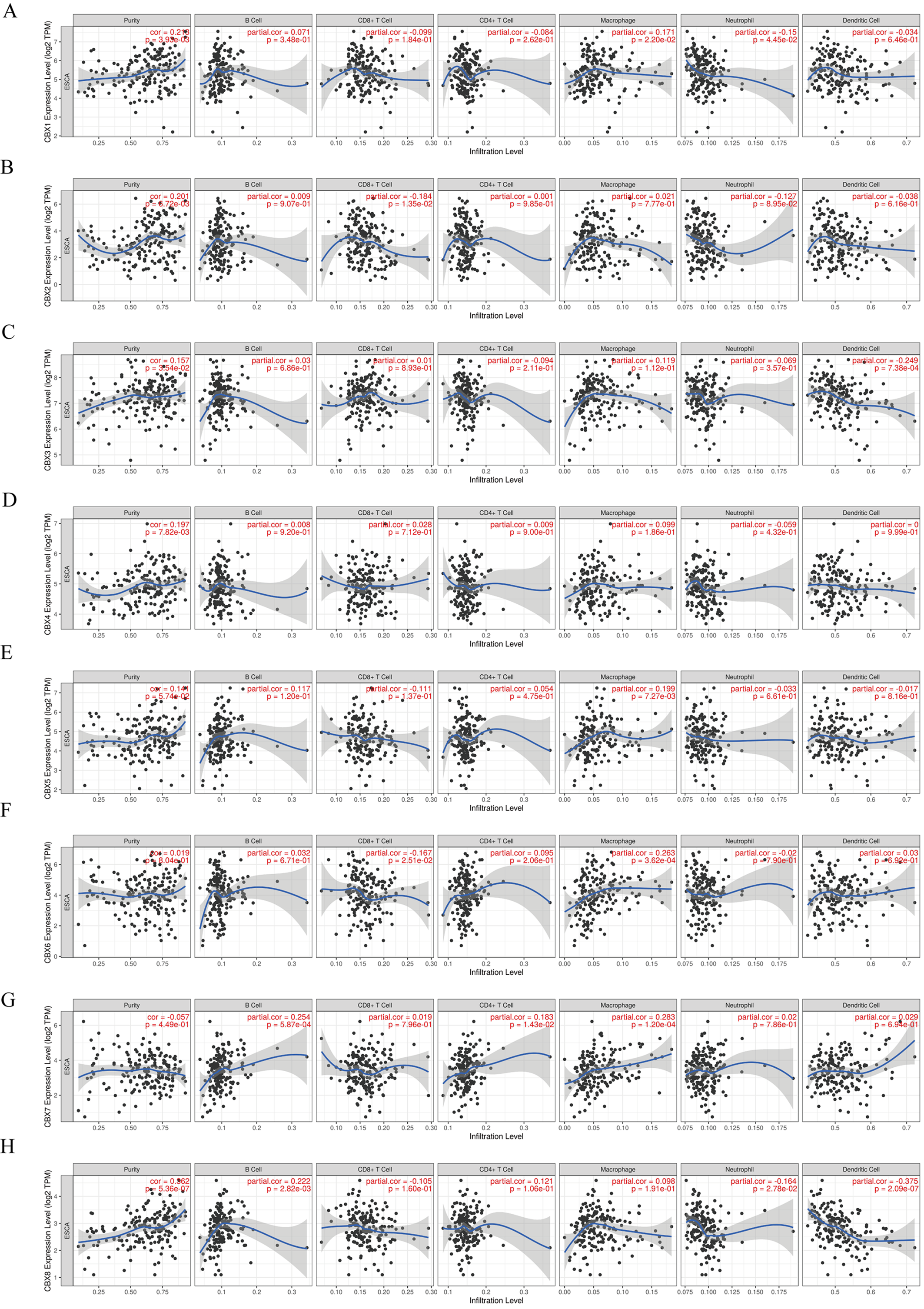

Supplement: Supplementary file 3 [file Image4.TIF]

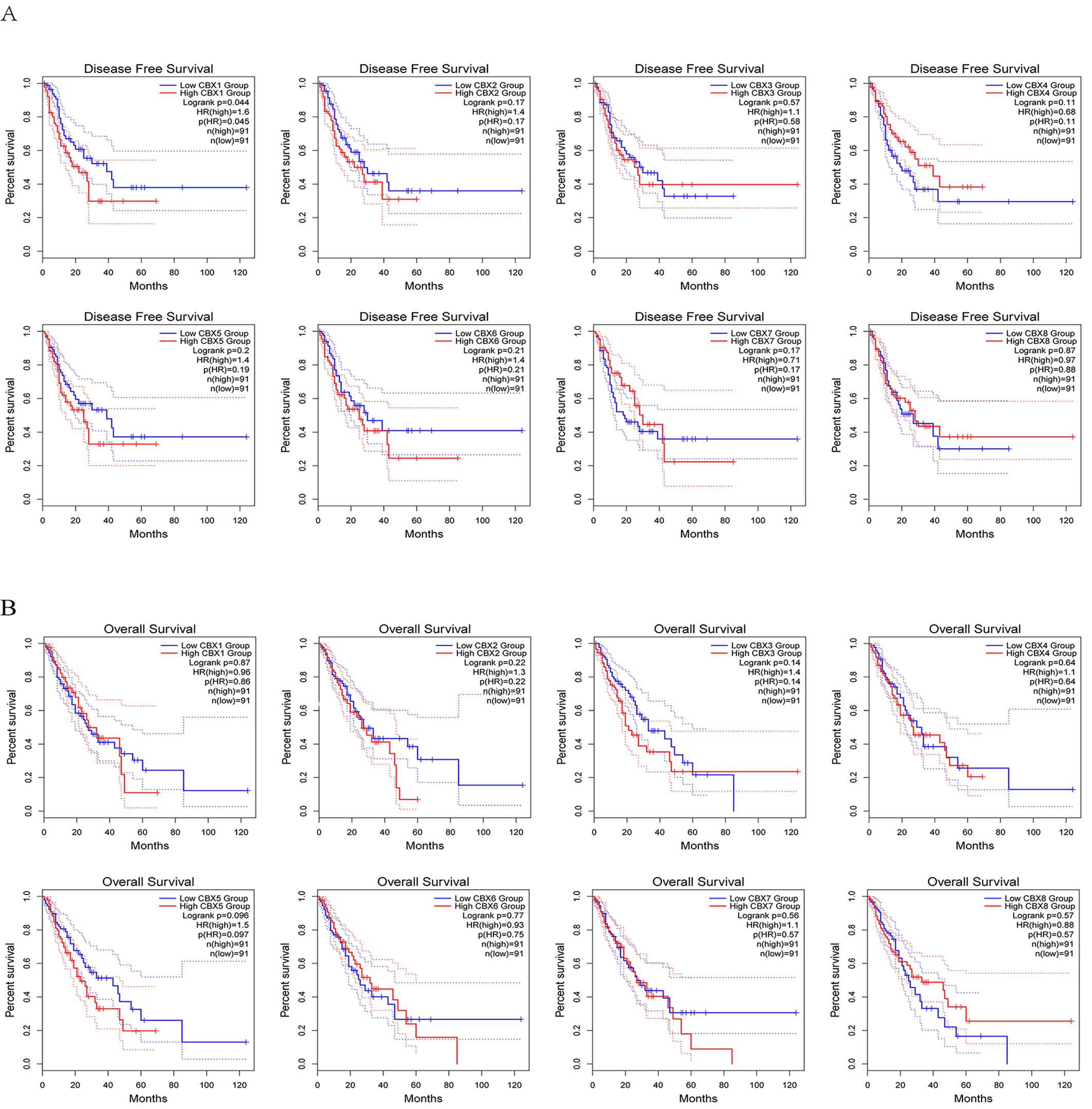

Supplement: Supplementary file 4 [file Image2.TIF]

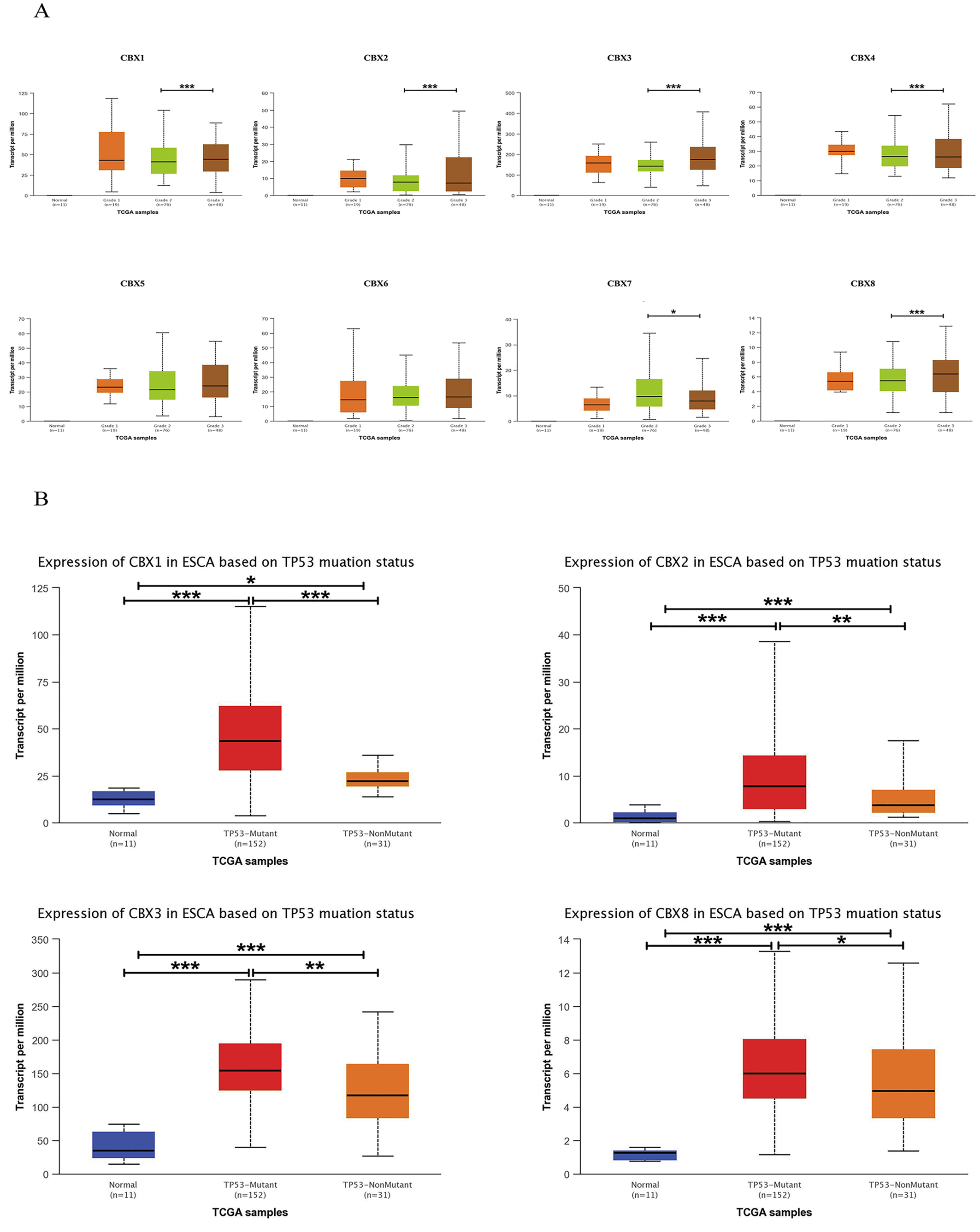

Supplement: Supplementary file 5 [file Image1.TIF]

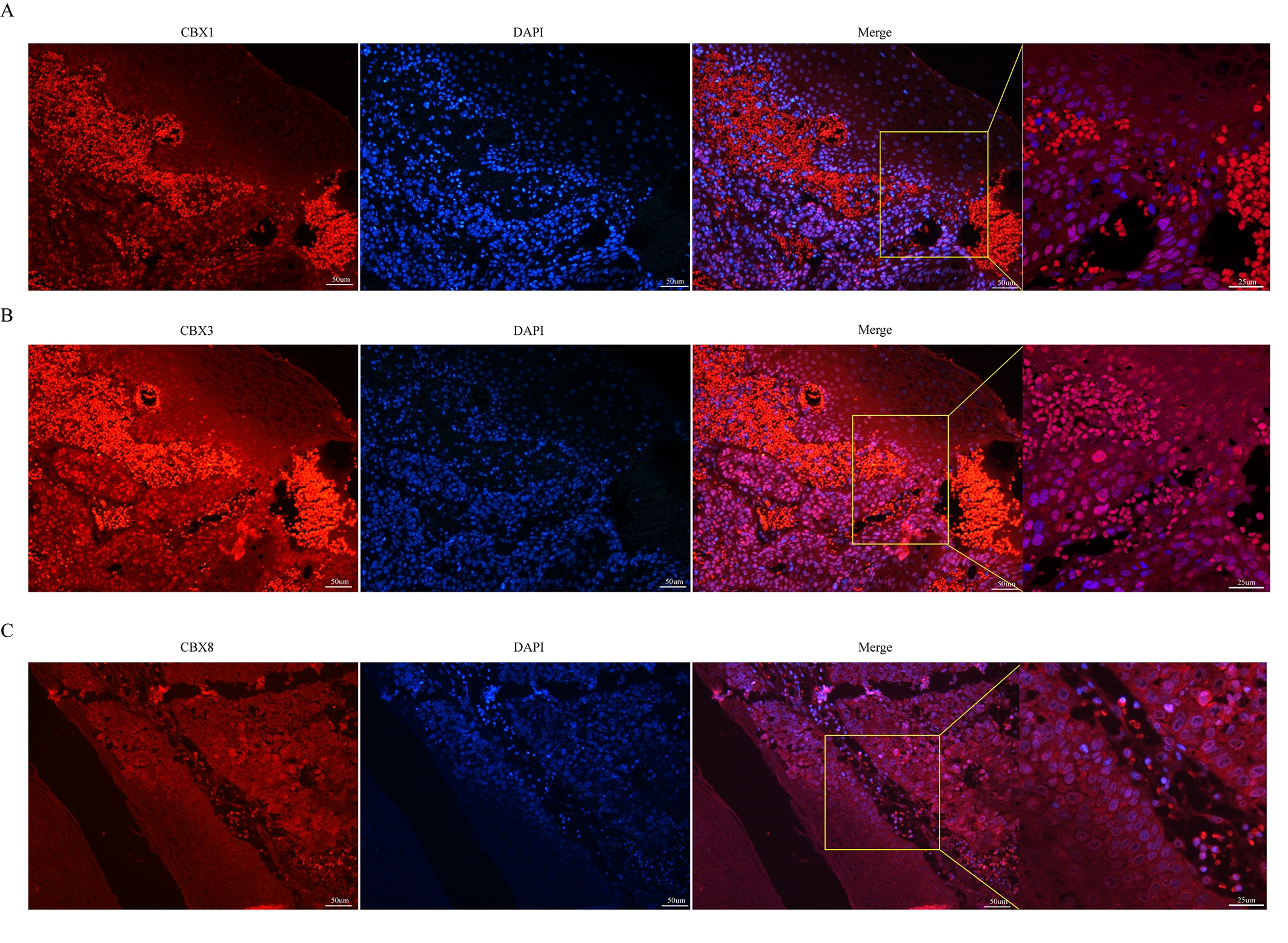

Supplement: Supplementary file 6 [file Image7.TIF]

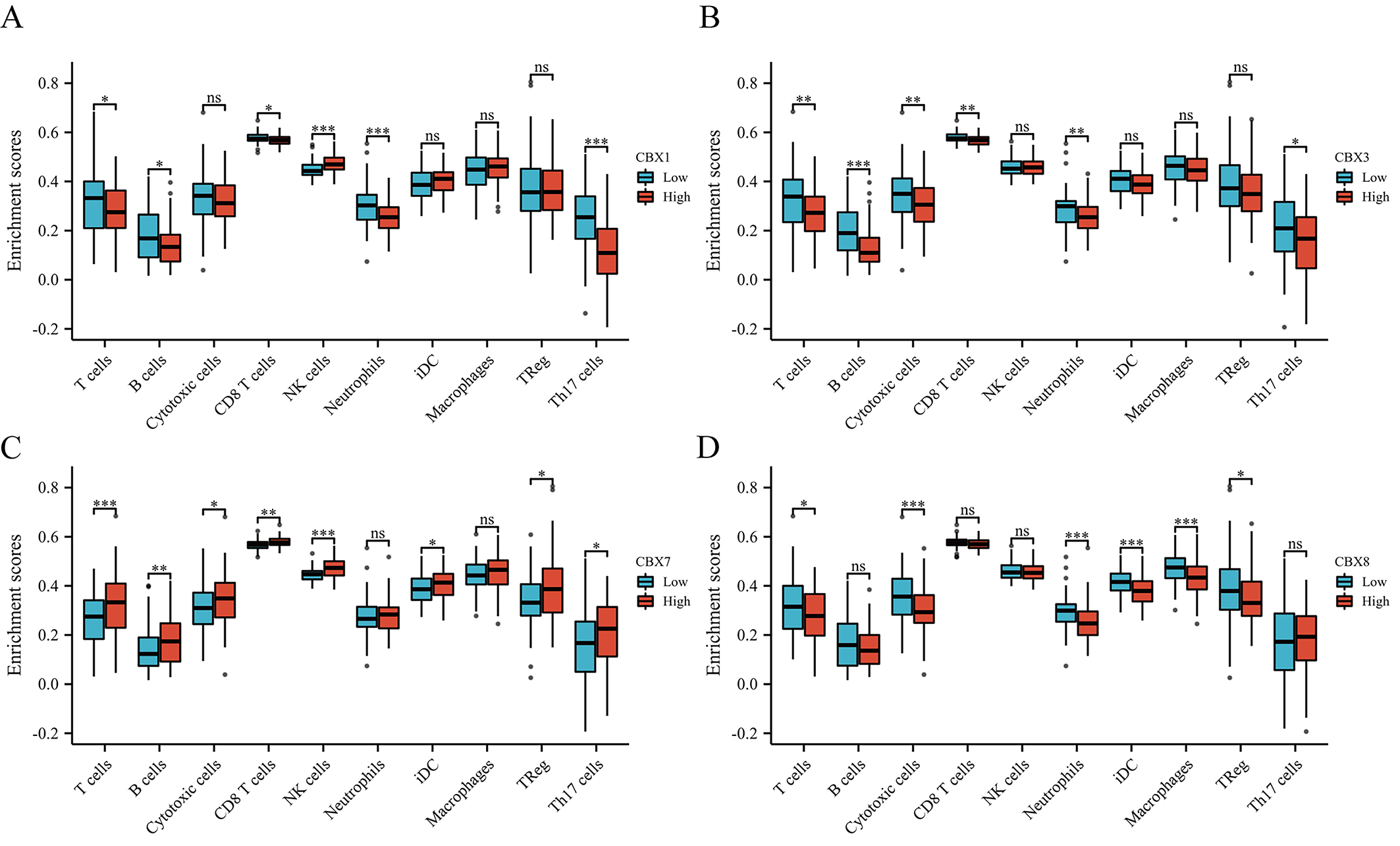

Supplement: Supplementary file 7 [file Image5.TIF]
