# Supplementary material for: Identification and Validation of Chromobox Family Members as Potential Prognostic Biomarkers and Therapeutic Targets for Human Esophageal Cancer
Source: Front Genet. 2022 Apr 6;13:851390. doi: 10.3389/fgene.2022.851390 (PMC9019303; doi:10.3389/fgene.2022.851390)
Supplement: Supplementary file 8 [file DataSheet1.docx]

**SUPPLEMENTARY IMFORMATION**

Supplementary Figure 1. (A) Association of mRNA expression of distinct CBX family members with tumor grades of ESCA patients. *p<0.05, **p<0.01, ***p<0.001.(B) Association of mRNA expression of distinct CBX family members with TP53 mutation status of ESCA patients. mRNA expressions of CBX1/CBX2/CBX3/CBX8 were significantly higher in TP53 mutation. *p<0.05, **p<0.01, ***p<0.001.

Supplementary Figure 2. The prognostic value of different expressed CBX family members in ESCA patients in the disease-free survival curve(A) and overall survival curve (B)(GEPIA).

Supplementary Figure 3. (A)The prognostic value of CBXs in ESCA patients in the overall survival curve (TCGA). (B) The prognostic value of CBXs in ESCA patients in the disease specific survival curve (TCGA). (C) The prognostic value of CBXs in ESCA patients in the progress free interval survival curve (TCGA).

Supplementary Figure 4. Correlations between differentially expressed CBX family members and immune cell infiltration (TIMER). Correlations between the abundance of immune cells and the expression of CBX1-8.

**Table S1.** PCR primers used in this study

| **Purpose** | **Forward primer (5´-)** | **Reverse primer (5´-)** |
| --- | --- | --- |
| *hACTB* expression | CCTGGCACCCAGCACAAT | GGGCCGGACTCGTCATAC |
| *hCBX1* expression | GGTGGAAAAAGTTCTCGACCG | CCCATGTGTTGTCCTCATCTG |
| *hCBX2*expression | GCCCAGCACTGGACAGAAC | CACTGTGACGGTGATGAGGTT |
| *hCBX3* expression | TAGATCGACGTGTAGTGAATGGG | TGTCTGTGGCACCAATTATTCTT |
| *hCBX4* expression | ACCGTGCCAAGCTGGATTT | AGGTCGTACATTTTGGGGTCG |
| *hCBX5* expression | AACAGTGCCGATGACATCAAA | GCCCCAATGATCTTTTCTGGT |
| *hCBX6* expression | ACCCAAACCCAAAACTTTCCT | GTCTCCGAGAAGGGCGAAAT |
| *hCBX7* expression | GCGTGCGGAAGGGTAAAGT | GCTTGGGTTTCGGACCTCTC |
| *hCBX8* expression | ATACGGAAAGGACGCATGGAA | CTTGGGTCCACGCTTTTTGG |
